# American Academy of Dental Science

**Published:** 1899-04

**Authors:** 

**Affiliations:** American Academy of Dental Science


### Reports of Society Meetings.

### AMERICAN ACADEMY OF DENTAL SCIENCE.

The regular monthly meeting of the American Academy of Dental Science was held at Young's Hotel, Wednesday evening, December 7, 1898, at six o'clock.

A paper was read by Dr. George T. Baker, of Boston, entitled "Formaldehyde in Dentistry."

(For Dr. Baker's paper, see page 221.)

*Dr. G. T. Baker.*—Thinking that the subject of formaldehyde might be a profitable one for discussion, I have prepared just a few words to read concerning it. Before reading, I will take this pastil, which is the gas in solid form, and by putting it in this cup, and then placing the cup over the lamp, and the lamp in the oven, the gas will be generated before I am through reading. One of these pastils contains five grains, which is sufficient to destroy any bacteria or disease germ in a cubic foot of space in ten minutes. This oven contains just one cubic foot.

This other apparatus is a formaldehyde generator. It generates the gas from wood alcohol. This lamp is filled with the alcohol, then inside this tube are two disks, and below are holes to allow the admission of air. When it is lighted, these disks become very hot, and the fumes of the wood alcohol, mixed with air passing up through the tube and over the incandescent disks, become converted into formaldehyde gas. Mr. Chase, of Melvin & Badger, tells me that this is probably the way in which all formaldehyde is manufactured in Germany and France, only on a very large scale, of course, but the operation is the same as that lamp.

In regard to this oven. I have for experiment put the lamp in there with no pastil in it, and have found that the supply of oxygen is just enough to keep the lamp burning for from six to fourteen minutes, depending on the height of the flame, and it is just about the same when the lamp contains a pastil and the gas is being generated.

DISCUSSION.

*President Cooke.*—We are all surely interested in this method of sterilization. The subject is now open for discussion. I would like to ask Dr. Baker to inform us what he uses the large lamp for?

*Dr. G. T. Baker.*—The large lamp is not used at all. I simply brought it for the purpose of showing how the gas is manufactured. Most, if not all, of the formaldehyde used in this country is manufactured in Germany and France, and this is the way it is done, —the fumes of the wood alcohol, mixed with air, pass over these heated disks and the result is formaldehyde gas.

*President Cooke.*—Could it be used for the fumigation of a room after a person had been sick in it?

*Dr. G. T. Baker.*—That is what it was designed for. There are a great many formaldehyde lamps on the market, but the lamp that uses the pastil is the best. The pastil, which is called paraform, is the gas in solid form, and by putting it in the lamp the heat sets free the gas and you get pure formaldehyde.

*President Cooke.*—What is the cost of an outfit like that?

*Dr. G. T. Baker.*—I think about $6.50. They have them at Metcalf's, Codman & Shurtleff's, and other dealers. I bought my oven and lamp at Bassett's, and it seems to work very nicely, and I put all sorts of instruments in the oven overnight,—clean them first and then sterilize them. Of course instruments to be sterilized must first be cleaned. They may be thoroughly cleaned by the use of soap and water, and still not be sterile. It is a deodorizer as well as a sterilizer, and the deodorizing property to me is fully as useful as anything else. Sometimes we want to get rid of odors; for instance, in the course of our work we are brought into close proximity with our patients, and supposing one person used a certain perfume very disagreeable to another, this can be readily overcome by deodorizing your office coat.

*Dr. Williams.*—It will clear a room of the odor of tobacco, and it seems to me an excellent thing for sterilizing the hand-piece of an engine. I do not know of any other way that it could be sterilized unless taken apart.

*Dr. Stevens.*—I was asking a man the other day, who was in my office explaining the merits of thycalol, why they did not use formalin for the antiseptic. He said it was no use putting it in, because it would evaporate, and would not remain in the solution. I wondered whether that was true. We have some solutions that claim to have formaldehyde in them, but whether it evaporates and leaves the residue I would like to know.

*Dr. Fillebrown.*—It will dissipate in a very short time. I use it a good deal in my case of instruments, saturating absorbent cotton with it, and it will fill that case with formalin gas, but it will not be very long before the cotton will be dry.

*Dr. Stevens.*—I should expect this result; but suppose it was in a solution, and kept corked as solutions usually are, will the formaldehyde evaporate?

*Dr. G. T. Baker.*—It will stay in a solution if proper care is taken. If put on cotton or instruments, I should not be surprised if it did evaporate very quickly, as it is very diffusible, but I know that it will keep its strength in a solution in a bottle for a long time.

*Dr. Maxfield.*—How can you test whether it has lost its strength or not?

*Dr. G. T. Baker.*—You can test its strength by the taste or smell. It makes a valuable thing to use as the antiseptic element of a mouth-wash. As a guide in making up solutions, I would say that ten drops of the forty-per-cent. solution to an ounce of water makes about a one-half-per-cent. solution formaldehyde, and this is strong enough ordinarily for the mouth, while for a gargle for the throat a much weaker solution should be used.

For sterilizing instruments the gas is to be preferred to the solutions.

*Dr. Taft.*—Do I understand that you use that after every operation?

*Dr. G. T. Baker.*—Every time you want to be sure that you have a sterile instrument. I make a practice of putting my instruments and office coat in the oven and leaving them overnight.

*Dr. Taft.*—What do you do about your hands?

*Dr. G. T. Baker.*—It is a splendid deodorizer. For instance, if you have been treating a pulp-canal, a one-per-cent. solution will deodorize your fingers perfectly; it practically has no odor of its own.

*Dr. Taft.*—Is it not just as important that you sterilize your hands after each operation as well as your instruments?

*Dr. G. T. Baker.*—You can do that, if you wish.

*Dr. Taft.*—Do you do that after each operation?

*Dr. G. T. Baker.*—No; I do not, as a rule. I simply wash my hands thoroughly in soap and water, occasionally using formaldehyde for deodorizing.

*Dr. Taft.*—I do not see why soap and water will not do for the instruments just as well.

*Dr. G. T. Baker.*—Hot water will, if you leave them there long enough.

*Dr. Werner.*—I would like to ask Dr. Baker,—for I think this is a very practical and nice thing for us to know, and it is a subject which we should not attempt to ridicule,—I would like to know what the disks in the funnel of the large apparatus are made of?

*Dr. G. T. Baker.*—They are made of copper.

*Dr. Werner.*—This matter was incidentally referred to before in our Academy during the discussion of another subject, and, if I remember correctly, the gas was generated with a funnel, or chimney, in which the disks were platinized asbestos. Is this an improvement over that method?

*Dr. G. T. Baker.*—I cannot say. I am only familiar with the method that generates the gas by heating the pastils over an alcohol lamp. Drs. Reik and Watson, and Mr. Morton, of the bacteriological department of the University, who have studied and written upon this subject,—all three of those gentlemen say that this is the most perfect method yet devised for generating pure formaldehyde gas.

*Dr. Eames.*—I am in hearty sympathy with what has been said this evening in regard to this sterilizer, but there are some things to be said regarding it, as a new thing, not entirely settled. Are we not carrying organisms into the mouth quite as well by means of bibulous paper or cotton, as by instruments or hands? Certainly, to be truly surgical, the hands, the absorbent cotton, the bibulous paper, or anything that is put in the mouth, should be thoroughly aseptic. It is a question whether boiling is not the surest sterilizer for those instruments or those materials which can be boiled. With the mouth-mirror and the right angle of the engine, there is a question whether it is not best to immerse them in a solution rather than to subject them to the formaldehyde gas. A recent German paper makes the statement, and no doubt it has been verified by others in this country, that if there is a layer of moisture on the instruments when they are put in a sterilizer of this kind, it fails to do the work. Last evening I was present at a meeting of the Metropolitan Society, and Dr. Lamkin, of Lynn, presented a sterilizer which he had modified from an ordinary tin cake-chest. This, with the lamp at one dollar and a half, makes a sterilizer which costs only one-third the price of the one shown this evening. This modification, if successful, will be of interest to dentists generally.

*Dr. Belyea.*—I have tried the cake-box with the formaldehyde gas for sterilizing. The only trouble I had with it was the rusting of some of my instruments. Possibly that was caused by a leakage of air, the cake-box, of course, not being thoroughly air-tight.

*Dr. Potter.*—I am very glad to see this question of sterilization brought out. It is one in which I have for a long time taken a great deal of interest. Personally, I prefer to boil my instruments in order to sterilize them. Still, I recognize the fact that there may be other ways of accomplishing the same result. For the benefit of those who boil their instruments, I wish to mention a boiler which I found this summer and which I have used with a good deal of satisfaction. It is an enamelled steel asparagus dish fitted with a perforated tray. Instruments can be placed on the tray and then immersed in the boiling solution. These dishes can be bought at F. A. Walker & Co.'s, 85 Cornhill, Boston. With regard to formaldehyde, I have been watching the literature for some time. You know very well the extravagant claims that were made for it when it first came out. It was said that formaldehyde gas would search out bacteria, even when wrapped in blankets and hidden away in mattresses. Late experiments show that formaldehyde gas will not reach bacteria so placed except in a vacuum chamber. It has also been very clearly shown that formaldehyde gas does not penetrate moist substances. There are, therefore, limitations in the use of formaldehyde gas which cause one to hesitate before adopting it as a sterilizing agent. And yet it may prove to be the most desirable means which we have of sterilizing instruments. I am looking forward to it with a great deal of expectation, knowing that there are undesirable things connected with heat sterilization which I would be glad to get rid of by the adoption of some other method if I could feel certain that the new method would accomplish the desired result.

I would like to refer the Academy to an excellent paper by Dr. Charles Harrington, of the Harvard Medical School, entitled "Possibilities and Limitations of Formaldehyde as a Disinfectant," published in the *American Journal of the Medical Sciences*, January, 1898.

*Dr. Werner.*—Will the essayist please tell us the solution of formalin he uses on his hands that he thinks is strong enough as a sterilizer and deodorizer and yet would not be injurious to the skin?

*Dr. G. T. Baker.*—I think it is a question as to the exact strength that is required. The different authorities give a slight difference as to what strength should be used. Mr. Bird says 1: 250 formaldehyde for general disinfectant solution for washing hands, sterilizing instruments, etc. That would be equivalent to a 1: 100 of the forty-per-cent. solution, or one per cent. I do not think it makes any great difference. In my use of it I put about one teaspoonful of the forty-per-cent. solution into the bowl, containing about one pint of water, which makes about one-half per cent. formaldehyde solution, stronger, perhaps, than need be, but it does not affect the hands, though it does deodorize them, and according to the authorities it does sterilize them.

*Dr. Werner.*—I think that is practical information. My difficulty is in deodorizing my hands after cases of pyorrhœa alveolaris and putrescent pulps. They are the most disagreeable odors to get rid of. You cannot do it by merely washing your hands in soap and water, unless by the addition of something else to disguise it, which may be equally as disagreeable to the patient.

*Dr. Fillebrown.*—I wish to say a few words to commend formalin to the attention of those who have not used it in connection with the care of their instruments. I was fortunate in obtaining a package from the first importation that was made from Germany. Dr. Jack spoke to me about it, and I have used it constantly ever since. The directions for its use are essentially the same as they were at that time,—that is, that one-half of one per cent. was sufficient for all ordinary cleansing purposes. It takes the place of bichloride, 1: 1000, and of course of anything weaker. It is unnecessary, I think, in an ordinary dental practice to sterilize the instruments more than once in twenty-four hours, and if you have a solution 1: 100 of formalin that your instruments are washed in after using them, you will have them thoroughly aseptic during the day. Formalin does not rust the instruments and keeps them perfectly clean. I have one of the sterilizers, and I find it an excellent thing to put in many of my instruments, napkins, sponges, and leave them there overnight, and they are all ready for the day's work. It does them no harm to leave them there. I have allowed instruments to remain two days in the sterilizer and when I took them out they were in perfect condition.

Professor Ernst thinks that if instruments having no grooves are washed clean in a 1: 100 solution and put in a case in which I keep a piece of cotton saturated with the formalin, it is abundantly sufficient to destroy all the germs on them. It being so easily used, I think it is a very valuable thing.

Speaking of the strength which could be used in washing of hands without injury, I had the temerity when it first came out to wash my hands in the full strength,—that is, forty per cent. formaldehyde. There was no great harm done, although it was a little rough on the cuticle; but weaken this with four or five times its quantity of water, and you can use it with perfect impunity on the skin.

*Dr. Maxfield.*—There is one other point I would like to see brought out to get the experience of the gentlemen present. You will remember that when formalin was first introduced as a disinfectant Dr. Howe experimented with it in the treatment of pulpless teeth, and he reported to the Odontological Society of New York several cases where he had been unable to close the canal when using other antiseptics, but by using formalin in the canal he was able to close it with no trouble; that is to say, in those cases an irritation had been set up within ten hours after closing the cavity where some well-known antiseptic had been used, but after using the formalin no further trouble occurred. Now, my experience in that way has not been very encouraging, for it has seemed to me in my use of it in pulp-canals that it has acted more or less as an irritant; the gas seemed to penetrate through to the end of the root and cause considerable pain, and pain that was not easily controlled. If the members would state what their experience has been in such cases, I would be very glad to hear it.

*Dr. Allen.*—I can add to Dr. Maxfield's testimony on that line. I have reason to believe that formalin caused very severe pericementitis where I applied it a few weeks ago, and it was not easily controlled. I used it as a disinfectant in a cavity before filling.

*Dr. Williams.*—Mr. President, I have had several instances of that sort, and on the general principle that formalin is an irritant, we should be very conservative in its use. With that knowledge, I avoid putting it into the ends of roots that are open. If there has been some previous disinfection and the end of the root is stopped, then of course you can sterilize the rest, if you please, with formalin. But, although readily effective, I have also found that it is not a durable disinfectant. It is rapidly dissipated, and unless there was some sort of reservoir to hold the supply, I should not want to depend upon it for permanent disinfection. It seems to me that a more permanent effect could be obtained by the use of some of the phenols, but if a person should wish to use formalin, I would emphasize the importance of avoiding making applications where it may get access to the vital tissues.

*Dr. Maxfield.*—I would say that I used a two-per-cent. solution in the canals in the same manner as described by Dr. Howe.

*Dr. Williams.*—To carry out a little analogy of the thing: You know of course the essential principle of this is formic acid, which is the same as the bee puts an atom of into the honey to preserve it. If the honey is taken out of the comb, the formic acid evaporates and the honey changes in appearance, and we know how the sting of the bee evaporates; so this is practically the same principle,—we are simply using the poison of the sting of the bee in a dilute form.

*Dr. Brackett.*—My experience with formaldehyde has been, I might say, superficial, from the fact that I have made no special study of the subject, and yet it seems not to have been in accord with those of the gentlemen who have just been speaking. It was first brought specially to my attention by Professor Wolcott Gibbs. A few years ago he brought to my office a one-half of one-per-cent. solution. That solution was the strongest that I had as a starting-point, and diluting that somewhat, so as not to be at all unpleasant to the taste, I used it in washing out alveolar abscesses and for various cleansing and sterilizing purposes without ever noticing any such results as the speakers have just mentioned. The dilution was made without accuracy, but probably the solutions used in my syringing out of alveolar abscesses and for similar cleansing purposes were not stronger than from one-twentieth of one per cent. to one-tenth of one per cent. I have also used these weak solutions for dipping the cotton which I have used as dressings in pulp-canals, and also as an antiseptic in cases of pyorrhœa alveolaris. I have no memory of any serious after-pain resulting; a little transitory smarting is the most serious that I remember. In my use of it, it did impress me as a valuable agent for all the objects that I have mentioned, but the dilution was quite considerable.

*Dr. Williams.*—You will notice that Dr. Brackett says that even with these very weak solutions there were some cases of transitory irritation.

*Dr. Brackett.*—It could hardly be called an irritation,—just a brief smarting, such as might come from the application of alcohol. I do not remember any intractable pain or anything that persisted for more than a very short time.

*Dr. G. T. Baker.*—One point has not been spoken of to-night,—that is, the use of formaldehyde in teeth with live pulps. It is a great irritant, and should be used in very weak solutions, and in teeth with live pulps I do not think that it should be used at all. I have never had any experience in using it that way, but I have heard that it will destroy the pulp just as arsenic will, but in pulpless teeth I think that it makes an excellent disinfectant if used in sufficiently weak solutions. I know the first time I used it for the disinfecting of a pulpless tooth, I used about a one-per-cent. solution, and the patient came back, complaining that the tooth was aching, so after that I used weaker solutions, and have never had any trouble since. The solutions of formaldehyde are not recommended for the sterilizing of instruments, as it has been found that the gas itself can be used more conveniently and will sterilize them perfectly, according to the testimony of eminent bacteriologists, and they all agree that it is one of the most powerful germicides known. Dr. Peck, of Chicago, lately made some experiments to determine the comparative strength of solutions of formaldehyde and bichloride of mercury, and he found that one in one thousand solution of bichloride of mercury was three to four times as powerful as a one in one thousand solution of formaldehyde. He also showed that the concentrated solutions of formaldehyde were very irritating and should be handled with due care.

HARRY E. CUTTER, D.D.S.,
*Editor American Academy of Dental Science.*

